# Enablers and Barriers to Home Management for Children with Gastroenteritis: Systematic Review

**DOI:** 10.1016/j.jpedcp.2024.200115

**Published:** 2024-05-15

**Authors:** Anouk A.H. Weghorst, Joanna Lawrence, Danielle E.M.C. Jansen, Gea A. Holtman, Lena A. Sanci, Marjolein Y. Berger, Harriet Hiscock

**Affiliations:** 1 Department of Primary and Long-Term Care, University of Groningen, University Medical Centre Groningen, Groningen, The Netherlands; 2 Department of Paediatrics, University of Melbourne, Parkville, Victoria, Australia; 3 Health Services Research Group, Murdoch Children’s Research Institute, Melbourne, Victoria, Australia; 4 Department of General Practice, University of Melbourne, Parkville, Victoria, Australia

## Abstract

**Objective:**

To identify enablers and barriers to home management for children with acute gastroenteritis perceived by health care professionals and caregivers.

**Study design:**

A systematic review was conducted using the following databases: PubMed, Embase, Web of Science, and Cumulative Index to Nursing & Allied Health. Studies from high-income countries published from 2003 to 2023 who included children with acute gastroenteritis younger than 6 years, treated via home management, and addressed enablers or barriers from the perspective of healthcare professionals or caregivers, were eligible for inclusion. Studies were independently reviewed for inclusion, data extraction, and quality assessment. Data synthesis was conducted using the Theoretical Domains Framework and Capability, Opportunity, Motivation-Behavior model.

**Results:**

In total, 4476 studies were screened, with 16 meeting the inclusion criteria. The commonest enablers for health care professionals concerned the “opportunity” component (ie, access to clinical decision tools, protocols, provision of free oral rehydration therapy), followed by their “capability” component (ie, knowledge about guidelines, oral rehydration therapy, and ondansetron) to initiate home management. Conversely, caregivers’ factors relied more on internal factors within the “motivation” component (ie, emotions, insecurity, need for reassurance), whereas “opportunity” components (ie, information sheets, monitoring calls) could assist them in managing their child with gastroenteritis at home.

**Conclusions:**

Health care professionals could benefit from enhanced capabilities and clinical decision support systems, whereas caregivers may require access to information resources and support for positive emotions and beliefs in their capabilities. Addressing these aspects could optimize home management, potentially allowing more children with acute gastroenteritis to be treated at home.

Acute gastroenteritis is one of the most common childhood diseases and can be effectively managed at home in children aged older than 6 months.^1^^,^^2^ Especially in high-income countries, where most children present without severe dehydration, effective home management can reduce the burden of gastroenteritis on children and the health care system.^2^, ^3^, ^4^ However, home management remains suboptimal.^3^, ^4^, ^5^

Optimal home management for children with acute gastroenteritis involves preventing dehydration through symptom monitoring, adequate rehydration, and the use of oral rehydration therapy (ORT), with ondansetron if needed.^1^ Early home-administered ORT can reduce complications, health care visits, and hospitalizations,^5^^,^^6^ but it remains underused in high-income countries.^7^ Caregivers play a vital role in appropriate home management, either with or without the intervention of a health care professional who can guide them in management.^5^^,^^8^ In 2003, an overview of factors influencing ORT revealed barriers including parental and health care professionals’ knowledge deficits, cultural practices, preferences for intravenous rehydration therapy, and the perception that vomiting contraindicates ORT.^9^ However, in recent years, management approaches have changed, with the implementation of ondansetron—an antiemetic medication—supporting home management. Oral ondansetron is now recommended in addition to ORT for children with increased risk of dehydration caused by vomiting.^10^, ^11^, ^12^ An overview of current data on enablers and barriers to home management for children with acute gastroenteritis, from the perspective of the 2 most important stakeholders, health care professionals and caregivers, is lacking.

Understanding the enablers and barriers and mapping them to theoretical mechanisms of behavior change may help identify tailored, effective approaches for increasing home management.^13^ Therefore, we aimed to systematically review the published literature on enablers and barriers to home management for children with acute gastroenteritis, from the perspective of health care professionals and caregivers.

## Methods

### Design

This systematic review was conducted according to the Preferred Reporting Items for Systematic Reviews and Meta-analysis guidelines.^14^ The study protocol was developed a priori and registered in the International Prospective Register of Systematic Reviews on April 9, 2023 (CRD42023412777).

### Literature Search

A systematic literature search was performed with the input of medical librarians by using the following databases: PubMed, Embase via Ovid, Web of Science, and Cumulative Index to Nursing & Allied Health. The search strategy was piloted and peer-reviewed by all authors. It was adapted to each specific database and performed on April 10, 2023 (Appendix, online; available at www.jpeds.com). The search included peer-reviewed studies published in the last 20 years, written in languages known to the research team (English, Dutch, German, French).

### Study Selection

Results from database searches were exported to Covidence and duplicates were removed.^15^ Inclusion criteria were (1) children with acute gastroenteritis younger than 6 years, (2) treated via home management, (3) addressed enablers or barriers from the perspective of health care professionals or caregivers, and (4) conducted in high-income countries, as defined by the World Bank.^16^ Studies reporting only data of children admitted to hospital were excluded. Single case reports, protocols, guidelines, opinions, book reviews, and conference abstracts also were excluded. Extraction of title and abstracts, followed by full-text screening was independently performed by 2 authors. Disagreements were resolved through discussion and within the research group. The reference lists of all included studies were screened for relevant studies.

### Data Extraction and Quality Assessment

Data including aim, study design and methods, health care professionals’ or caregivers’ perspectives, child characteristics, and enablers or barriers were extracted independently by 2 authors and recoded on an extraction template in Covidence. The quality of included studies was assessed independently by the same 2 authors using the standardized critical appraisal instruments from the Joanna Briggs Institute Critical Appraisal Tools for each specific study design.^17^, ^18^, ^19^, ^20^ Questions were scored as yes, no, unclear, or not applicable. The risk of bias of individual studies was determined with the following cutoffs: low risk of bias if 70% of answers scored yes, moderate risk of bias if 50% to 69% of answers scored yes, and high risk of bias if yes scores were less than 50%.^21^

### Synthesis of Data: TDF and COM-B Model

**Figure 1:**
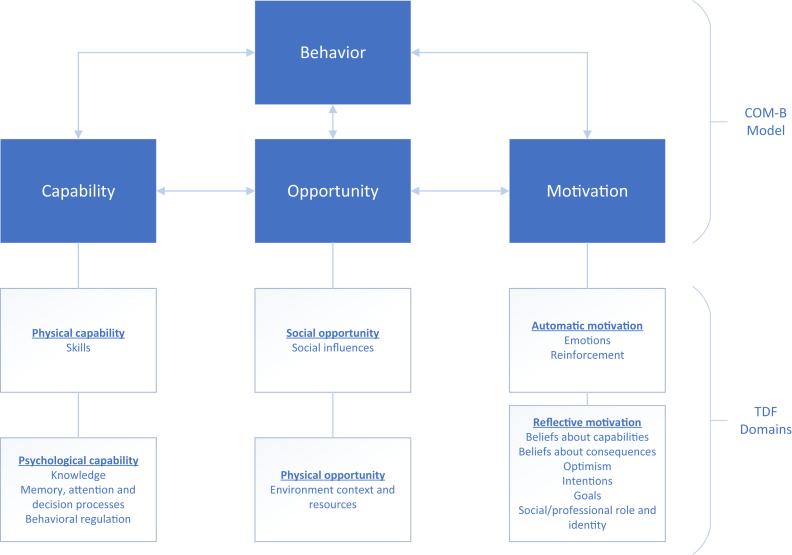
TDF and COM-B model.

In the absence of data to numerically quantify the effect of factors on health care professionals’ and caregivers’ behavior required to support home management, we used the Theoretical Domains Framework (TDF) and the Capability, Opportunity, Motivation – Behavior (COM-B) model instead to identify key enablers and barriers. The TDF integrates 14 theoretical domains derived from 33 behavior change theories and 84 theoretical constructs, providing a systematic and theory-based approach for identifying individual, social, and environmental influences on behavior (Table I).^13^^,^^22^ The 14 domains of the TDF can be consolidated into the COM-B model consisting of 3 fundamental components: capability, opportunity, and motivation (Figure 1).^23^ In the COM-B model, behavior (B) emerges from the interplay between psychological and physical capabilities (C), the use of physical and social opportunities (O), driven by either reflective or automatic motivators (M). The COM-B model delineates the necessary shifts for achieving desired behavior, thus guiding intervention targeting. Two authors independently mapped the extracted enablers and barriers on the TDF. Subsequently, the TDF domains were categorized into the 3 fundamental components of the COM-B model.

**Table I:** Theoretical domains framework^13^

TDF domains	Definitions
Knowledge	An awareness of the existence of something
Skills	An ability or proficiency acquired through practice
Beliefs about capabilities	Acceptance of the truth, reality, or validity about an ability, talent, or facility that a person can put to constructive use
Beliefs about consequences	Acceptance of the truth, reality, or validity about outcomes of a behavior in a given situation
Optimism	The confidence that things will happen for the best or that desired goals will be attained
Intentions	A conscious decision to perform a behavior or a resolve to act in a certain way
Goals	Mental representations of outcomes or end states that an individual wants to achieve
Memory, attention, and decision processes	The ability to retain information, focus selectively on aspects of the environment, and choose between 2 or more alternatives
Emotion	A complex reaction pattern, involving experiential, behavioral, and physiological elements, by which the individual attempts to deal with a personally significant matter or event
Behavioral regulation	Anything aimed at managing or changing objectively observed or measured actions
Social/professional role and identity	A coherent set of behaviors and displayed personal qualities of an individual in a social or work setting
Social influences	Those interpersonal processes that can cause individuals to change their thoughts, feelings, or behaviors
Environmental context and resources	Any circumstance of a person's situation or environment that discourages or encourages the development of skills and abilities, independence, social competence, and adaptive behavior
Reinforcement	Increasing the probability of a response by arranging a dependent relationship, or contingency, between the response and a given stimulus

Atkins et al.^13^

## Results

### Characteristics of Included Studies

**Figure 2:**
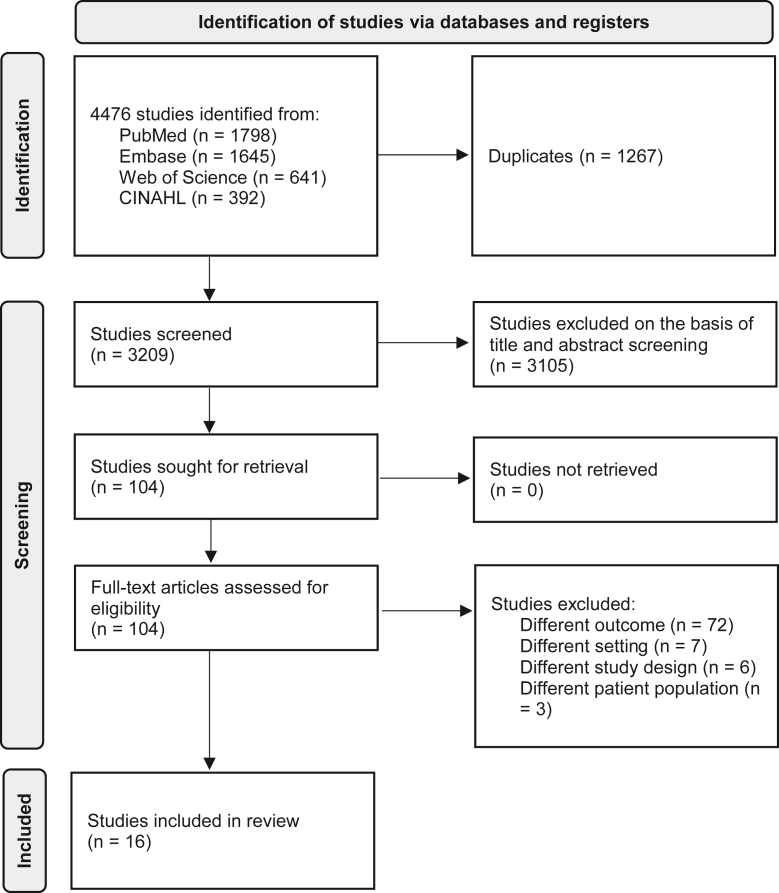
Study flow diagram. *CINHAL*, Cumulative Index to Nursing & Allied Health.^24^

The search strategy yielded 4476 records of which 104 were reviewed in full text, 16 met the inclusion criteria and were included in the analysis (Figure 2).^24^ Of these studies, 8 reported health care professionals’ (primary care pediatricians, pediatric emergency medicine physicians, and emergency department nurses)^25^, ^26^, ^27^, ^28^, ^29^, ^30^, ^31^, ^32^ and 8 reported caregivers’^33^, ^34^, ^35^, ^36^, ^37^, ^38^, ^39^, ^40^ perspectives. In total, 13 reported quantitative and 3qualitative results. A summary of study characteristics is presented in Table II.

**Table II:** Characteristics of included studies (n = 16)

Authors, year of publication	Country	Aim	Study design and methods	Study population	Characteristic of included children
Albano et al, 2010^25^	Italy	To evaluate the applicability and efficacy of guidelines for managing acute gastroenteritis in pediatricians undergoing specific training.	Randomized controlled trial. Intervention: 2-hour course about recommendations in the guidelines for acute gastroenteritis.	150 primary care pediatricians.	1309 children, 17 months (mean), mild-to-moderate dehydration.
Albrecht et al, 2017^33^	Canada	To describe caregivers’ experiences of pediatric acute gastroenteritis and identify their information needs, preferences, and priorities.	Qualitative study. Methods: semistructured interviews.	15 caregivers.	15 children, <5 years (93%).
Bahm et al, 2016^26^	Canada	To evaluate the impact of clinical decision tools in pediatric acute gastroenteritis.	Retrospective cohort study. Methods: linked survey data.	Pediatric emergency medicine physicians.	57 921 children, 5.0 years (mean).
Bender et al, 2007^27^	United States	To examine attitudes toward the use of oral rehydration therapy by pediatric emergency medicine physicians before and after being given recent data about guidelines.	Nonrandomized interventional study. Intervention: data refuting the barriers to the use of oral rehydration therapy.	445 pediatric emergency medicine physicians.	3 scenarios: child <2 years with mild, moderate or severe dehydration.
Eriksson et al, 2015^34^	Sweden	To describe parents’ experiences of monitoring calls in telephone advice nursing, in children with gastroenteritis.	Qualitative study. Methods: in-depth interviews.	10 caregivers.	10 children, aged 8-23 months.
Freedman et al, 2008^35^	Canada	To describe the reliability and validity of a caregiver gastroenteritis knowledge questionnaire and to identify specific knowledge deficits.	Analytical cross-sectional study. Methods: questionnaire with 38 true/false questions.	80 caregivers.	80 children, 3 months to 4 years.
Freedman et al, 2011^28^	Canada and United States	To examine practices, knowledge, and beliefs regarding the treatment of toddlers and young children with acute gastroenteritis in the emergency department.	Analytical cross-sectional study. Methods: online survey.	324 pediatric emergency medicine physicians.	Scenario about toddlers and young children with acute gastroenteritis.
Geurts et al, 2017^29^	Netherlands	To evaluate the feasibility and impact of a clinical decision support system for managing of children with acute gastroenteritis at the emergency department.	Randomized controlled trial. Intervention: nurse-guided clinical decision support system.	Emergency department nurses.	222 children, 1.4 years (median), mild-to-moderate dehydration.
Graham et al, 2010^36^	Canada	To examine parental motivations for bringing their child with symptoms of gastroenteritis to the emergency department.	Qualitative study. Methods: structured survey via telephone.	10 caregivers.	10 children, 3 months – 3 years.
Haines et al, 2012^37^	United States	To evaluate outcomes associated with a discharge action plan employing single-dose home use of ondansetron in patients with acute gastroenteritis.	Case series. Methods: clinical-conducted telephone call 3-5 days after emergency visit.	29 caregivers.	29 children, 5.15 years (mean).
Hendrickson et al, 2018^30^	Unites States	To determine whether a triage-based, nurse-initiated protocol for early provision of ondansetron and oral rehydration therapy could safely improve the care of children with gastroenteritis at the emergency department.	Non-randomized interventional study. Intervention: triage-based, nurse-initiated protocol.	Emergency department triage nurses.	128 children, 2.01 years (mean), no and mild dehydration.
Jové-Blanco et al, 2021^38^	Spain	To evaluate whether the addition of a video discharge instruction to usual verbal information improved the comprehension of information provided to caregivers of patients who consult in pediatric emergency department for acute gastroenteritis.	Randomized controlled trial. Intervention: video discharge instructions.	139 caregivers.	139 children, 2 years (median) 87.3% no, 9.3% mild, and 5% moderate dehydration.
Nicastro et al, 2015^31^	11 European countries	To assess the effect of a 5-module e-learning course about clinical practice guidelines for acute gastroenteritis on physicians' knowledge and clinical practice.	Nonrandomized interventional study. Intervention: e-learning course including 5 learning modules addressing the 5 key areas of acute gastroenteritis management.	149 physicians (95% pediatricians, 5% general practitioners)	545 children, 21 months (median).
Nir et al, 2013^39^	Israel	To evaluate parents’ attitudes toward rehydration methods used in pediatric emergency departments.	Analytical cross-sectional study. Methods: questionnaires.	100 caregivers.	100 children, >50% 0-3 years, mild-to-moderate dehydration.
Small et al, 2005^40^	Northern-Ireland	To compare clinical outcomes of admitted and home treated children with acute gastroenteritis presenting at Accident and Emergency Departments.	Prospective cohort study. Methods: medical records.	116 caregivers.	116 children, 1.85 years (mean).
Zolotor et al, 2007^32^	United States	To improve the quality and reduce cost associated with the care of gastroenteritis for children covered by Medicaid in the AccessCare network.	Non-randomized interventional study. Intervention: education sessions for healthcare professionals, free oral rehydration solution, patient education video, and feedback on oral rehydration use.	20 pediatric practices.	3367 children, <5 years.

### Quality of Studies

Most of the included studies (15/16) had a low risk of bias (Table III). For health care professionals, all enablers (11/11) and 80.0% (4/5) of barriers were derived from quantitative studies. Conversely, for caregivers, a smaller proportion of enablers (14.2%, 2/14) and barriers (1.7%, 3/28) related to home management were sourced from quantitative studies.

**Table III:** Quality assessment of the included articles

Articles	In- exclusion	Study sample	Validity exposure	Measurement condition	Confounders	Strategies for confounders	Validity measurement	Analysis
Analytical cross-sectional studies^19^								
Freedman et al, 2008^35^	Y	Y	Y	Y	Y	Y	Y	Y
Freedman et al, 2011^28^	Y	Y	Y	Y	N	N	Y	Y
Nir et al, 2013^39^	Y	Y	Y	Y	N	N	Y	Y

Cohort studies^19^	Group similarity	Exposures measured	Validity exposure	Confounders	Strategies for confounders	Free of outcome start	Validity measurement	Follow-up sufficient	Follow-up complete	Strategies incomplete follow-up	Analysis
Bahm et al, 2016^26^	Y	Y	Y	Y	Y	Y	Y	Y	Y	Y	Y
Small et al, 2005^40^	Y	Y	Y	Y	Y	NA	Y	Y	Y	Y	Y

Qualitative studies^18^	Perspective—methods	Methods—objectives	Methods —data collection	Methods—analysis	Methods—results	Researcher's background	Researcher's influence	Participants	Ethics	Conclusion
Albrecht et al, 2017^33^	Y	Y	Y	Y	Y	N	N	Y	Y	Y
Eriksson et al, 2015^34^	Y	Y	Y	Y	Y	Y	N	Y	Y	Y
Graham et al, 2010^36^	Y	Y	Y	Y	Y	N	Y	Y	Y	Y

Randomized controlled trials^17^	Randomization	Allocation concealed	Group similarity	Participants blinded	Delivering treatment blinded	Identical treated	Assessors blinded	Same outcomes measured	Reliable outcomes measured	Follow-up complete	Analysis allocated group	Analysis	Design
Albano et al, 2010^25^	Y	Y	Y	Y	N	Y	U	Y	Y	Y	Y	Y	Y
Geurts et al, 2017^29^	Y	Y	Y	U	N	Y	U	Y	Y	Y	Y	Y	Y
Jové-Blanco et al, 2021^38^	Y	Y	Y	N	N	Y	U	Y	Y	N	Y	Y	Y

Case series^4^	In-exclusion	Measurement condition	Validity methods	Consecutive inclusion	Complete inclusion	Participant's demographic	Reporting clinical information	Reporting outcomes	Reporting sites	Analysis
Haines et al, 2012^37^	Y	Y	Y	Y	Y	Y	Y	Y	N	Y

Nonrandomized interventional studies^20^	Cause and effect	Group similarity	Identical treated	Control group	Multiple measures	Follow-up complete	Same outcomes measured	Reliable outcomes measured	Analysis
Bender et al, 2007^27^	Y	Y	Y	N	Y	Y	Y	Y	Y
Hendrickson et al, 2018^30^	Y	N	Y	Y	Y	Y	Y	Y	Y
Nicastro et al, 2015^31^	Y	Y	Y	N	Y	Y	Y	Y	Y
Zolotor et al, 2007^32^	Y	Y	Y	N	Y	Y	Y	Y	Y

Yes, no, unclear, or not applicable.

### Enablers and Barriers: TDF

Enablers and barriers were categorized across 10 domains of the TDF (Table IV). Among health care professionals, 3 domains revealed enablers, whereas 4 domains revealed barriers. For caregivers, 6 domains indicated enablers and 9 domains indicated barriers to home management.

**Table IV:** Enablers and barriers relevant to health care professionals and caregiver for home management of children with acute gastroenteritis mapped to the TDF

TDF domains		Health care professionals	Caregivers
Knowledge	Enablers Barriers	• Knowledge of guidelines.^25^ • Knowledge of effectiveness of ORT and effect on length of stay.^27^ • E-learning about guideline management.^31^ • Lack of awareness of guidelines.^25^ • Lack of knowledge about ondansetron.^28^	• Learning about effective treatments.^33^ • New knowledge would impact their future actions and decisions.^33^ • Lack of understanding of signs and symptoms, course, and dehydration.^33^ • Misconceptions about home management.^33^ • Lack of knowledge for indications to see a physician, solid intake/refeeding, and medication use.^35^ • Lack of knowledge about treatment, etiology, signs, and degree of dehydration.^36^ • More likely to attend by first child.^36^ • Lack of knowledge about duration of symptoms.^37^
Skills	Enablers Barriers	• Nursing initiation of ORT.^26^^,^^32^ • Collaborating with other emergency departments.^32^ • Sites treating fewer children.^26^	• Exhausting own repertoire of treatments did not work.^36^
Beliefs about capabilities	Enablers Barriers		• Confirmation.^34^ • Share worries and responsibilities.^34^ • Getting positive feedback.^34^ • Multiple sick family members.^33^ • Primary caregiver for sick child and multiple children.^33^ • Illness out of keeping with their own expectations.^36^ • Hesitating without a medical opinion.^36^
Beliefs about consequences	Enablers Barriers	• Misbeliefs that ORT would increase length of stay.^27^	• ORT improved symptoms.^33^ • Child's symptoms not improving, worsening symptoms.^33^ • Nothing seemed to help.^33^ • Prolonged illness and worry about long-term consequences.^36^ • Parental perception of illness severity.^40^
Optimism	Barriers		• Magical place—kids always improve after visiting the emergency department.^36^
Intentions	Enablers Barriers		• Agree ORT if diarrhea.^39^ • Decline ORT if child is vomiting or refuses to drink.^39^ • Previous experience with similar illness requiring emergency care.^33^ • Previous dissatisfaction with telephone health advice service.^33^ • Previously intravenous treatment, tendency to not agree to ORT.^39^
Goals			
Memory, attention, and decision processes	Barriers	• Increased number of years in practice decreased change of ORT.^27^	
Emotions	Enablers Barriers		• Feeling of comfort, security, confidence and reassurance.^34^ • Being taken seriously.^34^ • Feeling of being important.^34^ • Higher parental stress.^40^ • Feeling scared, worried, uncertain, powerless.^33^ • Anxiety about etiology and alternate diagnosis.^36^ • Feeling helplessness when child is suffering.^36^
Behavioral regulation			
Social/professional role and identity			
Social influences	Barriers		• Advice from other parents, spouse or partner, day care staff, neighbors, and the internet.^36^
Environmental context and resources	Enablers Barriers	• Clinical decision tool with medical directive for ORT.^26^ • Free ORT distribution.^32^ • Nurse triage system for dehydration.^32^ • Protocol for ORT administration and monitoring.^32^ • Clinical decision support system.^29^ • Triage nurse-based protocol.^30^	• Use of information sheets from the hospital.^32^^,^^33^ • Video discharge instructions.^38^ • Monitoring calls.^34^ • Ondansetron provided before going home.^37^ • No formal, written information.^33^ • Latest technology in the emergency department.^33^ • Regular physician unavailable for advice.^33^^,^^36^
Reinforcement			

### COM-B Model

#### Capability

Capability refers to whether health care professionals and caregivers have the knowledge, skills, and abilities required for optimal home management (ie, preventing dehydration through symptom monitoring, adequate rehydration, and the use of ORT, with ondansetron if needed). The capability of health care professionals and caregivers strongly influenced their ability to manage their child with gastroenteritis at home.

Health care professionals who had more knowledge of acute gastroenteritis management guidelines and effectiveness of ORT were more inclined to adhere to recommended practices and to initiate ORT, serving as an enabler for home management.^25^^,^^27^^,^^31^ Designating an “ORT nurse” for staff training and collaborating with local emergency departments to advocate ORT use increased health care professionals’ skills to initiate home management.^26^^,^^32^ Conversely, health care professionals’ lack of knowledge about guidelines and clinical benefits of ondansetron posed a barrier to home management, evidenced by an increased use of nonrecommended interventions and decreased administration of ondansetron.^25^^,^^28^ In addition, lower skill levels among health care professionals correlated with greater revisit rates.^26^ Interestingly, health care professionals with more years of practice were more inclined to adhere to their own practices and less likely to incorporate ORT into management, serving as a barrier to home management.^27^

Caregivers with greater disease-related knowledge and experience tended to initiate fluid and ORT administration earlier, felt more confident in managing mild symptoms at home, and were more willing to wait longer before seeking emergency care.^33^ Conversely, first-time caregivers lacking knowledge and skills for appropriate home management were less likely to manage their child at home, underscoring the role of capability as a barrier to home management.^33^^,^^35^, ^36^, ^37^

#### Opportunity

Opportunity encompasses external factors, including physical and social influences, that shape behavior. For both health care professionals and caregivers, leveraging physical opportunities (ie, work place, processes, implementation tools or information resources) especially affected the home management.

For health care professionals, access to tools that increased their knowledge and skills (ie, a 2-hour course or e-learning) about gastroenteritis management increased the initiation of home management.^29^^,^^31^ Other tools that were important included the implementation of a clinical decision tool with medical directives for ORT, a clinical decision support system, a triage nurse-based protocol, a triage nurse system for dehydration, a protocol for ORT administration and monitoring, and a system to distribute free ORT during gastroenteritis visits.^26^^,^^29^^,^^30^^,^^32^ Implementation of a single tool resulted in increased appropriate ORT and ondansetron use,^26^^,^^29^^,^^30^ whereas implementation of multiple tools simultaneously not only increased ORT use but also reduced gastroenteritis admissions by 45%.^32^

For caregivers, access to resources, such as information sheets from hospitals, use of video discharge instructions, and monitoring calls from telephone nurses, enabled home management.^32^, ^33^, ^34^^,^^38^ Information sheets guided caregivers through necessary steps and aided in identifying signs of dehydration, whereas monitoring calls offered valuable additional information and opportunities to ask questions during various stages of the child’s illness at home. Moreover, providing ondansetron directly to caregivers, rather than issuing a prescription, enabled home management, and resulted in improved compliance and usage of ondansetron at home.^37^ Conversely, the lack of resources, such as written information provided to caregivers, created a barrier to home management, making it challenging for them to recall discharge and care instructions for current and future episodes.^33^ When health care professionals were unavailable for telephonic discussions or advice, caregivers were more inclined to visit the emergency department instead of managing the illness at home.^33^^,^^36^ Advice from other caregivers or daycare staff served as a barrier to home management, as the course of their child’s disease did not align with the information received from the surrounding environment.^36^

#### Motivation

Motivation refers to the internal processes and drives that influence decision-making and behavior, encompassing both conscious and unconscious processes. This component was particularly crucial for caregivers in their ability to manage their child with gastroenteritis at home, whereas it played a less significant role for health care professionals.

For health care professionals, beliefs about the consequences of ORT, such as potential prolonged emergency stays, reduced the likelihood of initiating ORT and acted as a barrier to home management.^27^ The motivations of caregivers were influenced by their capabilities and opportunities. Monitoring calls offered confirmation, support, feedback, and an opportunity to share worries, leading to increased confidence in managing the child at home.^34^ These calls also evoked positive emotions such as comfort, security, confidence, and reassurance, which increased the likelihood of caregivers treating their child at home.^34^ Specifically, regarding ORT, if the child accepted it well at the emergency department and when diarrhea was the main symptom, caregivers were more willing to continue ORT at home.^33^^,^^39^ Conversely, ORT use declined when the child presented with vomiting or refused to drink.^39^ Caregivers’ motivations also acted as barriers to home management. Concerns about prolonged illness, worries about long-term consequences, and an increased perception of illness severity were barriers for home management.^36^^,^^40^ Other emotions hindering home management included greater parental stress and feeling scared or worried about their child, which led to uncertainty about how to proceed with managing gastroenteritis at home.^33^^,^^40^ In addition, anxiety about potentially missing a serious condition, fear of alternative diagnoses, facing additional stressors (ie, having multiple sick family members or being the primary caregiver for multiple children), further prevented home management. Situations in which the child’s illness did not align with their expectations and hesitation to stay home without a medical opinion also posed barriers.^33^^,^^36^ In addition, previous negative experiences with telephone health services, ORT, or home management posed barriers.^33^^,^^39^

## Discussion

This is the first study that conducts a theoretical analysis of the potential enablers and barriers among health care professionals and caregivers in the home management of children with acute gastroenteritis. By applying the TDF and COM-B model, we identified key health professional and caregiver barriers and enablers to home management that should be considered in gastroenteritis interventions.

### Health Care Professionals

Almost all factors influencing the behavior of health care professionals were identified within the “capability” and “opportunity” components of the COM-B model. The greatest enablers for health care professionals pertain to their “opportunity” component, which is noteworthy, given that this component pertains to external factors enabling the execution of behavior. Various tools were found to initiate the home management for children with gastroenteritis with the provision of free ORT during gastroenteritis visits found to be particularly impactful. This finding aligns with previous research, indicating that offering ORT to families during their visits significantly enhanced ORT use and decreased unscheduled return visits.^41^ In addition, integrating multiple tools simultaneously had the most effect, as it not only increased ORT use but also reduced gastroenteritis admissions. Previous studies on practice changes revealed that combining multiple interventions changes produces better outcomes compared with single interventions.^42^^,^^43^

Some of the “opportunity” components influenced the “capability” of health care professionals, with access to tools (ie, a 2-hour course or e-learning) increasing their knowledge and skills. Moreover, health care professionals’ knowledge of guidelines and the efficacy of ORT enabled home management, reflected by increased guideline adherence and ORT prescription. This is consistent with research involving older children with gastroenteritis (average age 8 years), where educating medical trainees led to increased appropriate ORT and ondansetron use.^41^ Considering the hypothesized relationship between the components of COM-B, it is conceivable that as the “capability” and “opportunity” of healthcare professionals are improved, motivational factors, such as beliefs about the benefits of ORT and ondansetron, will be more widely applied in children with gastroenteritis at risk of dehydration.^44^

### Caregivers

Conversely, the most important factors influencing the behavior of caregivers were found in the “motivation” component, which refers to internal processes that influence behavior. Central to home management is the caregivers’ fear of missing something serious and concerns about the child’s safety.^36^ This review highlights the effect of negative emotions, such as stress, worry, uncertainty, and helplessness, acting as barriers to optimal home management, whereas positive emotions, including feelings of comfort, security and being taken seriously, enable home management. Previous research found that fears and concerns for childhood diseases often are influenced by personal experiences, stories from others, and information sourced from the internet.^45^^,^^46^ This emerges within the “opportunity” component, where the availability of appropriate resources enabled home management for caregivers. Bernhardt and Felter found that mothers, especially in the first few years after delivery, tend to be information seekers, especially on the internet.^47^ In this review, we found that resources providing information in various forms, such as video instructions, information sheets, and monitoring calls, are enablers for managing children with gastroenteritis at home. However, no effect was evaluated on the emergency department return visit rate. For childhood fever, caregivers who had access to an illness-focused interactive booklet on childhood fever had a significant reduction in their intention to reconsult for similar illnesses.^48^ It would be interesting to see what kind of information resource would enable home management the most for children with acute gastroenteritis and prevent returns to the emergency department. By evaluating information resources, it is important to keep in mind that only 61% of caregivers can identify more than one sign of dehydration and the definition of diarrhea is not completely understood.^49^ In this review, we found information deficits in various areas, including understanding etiology of the disease, recognizing signs and (alarm) symptoms (of dehydration), knowing management options, and determining when to seek professional help. This information should therefore be included in any information resources.

### Limitations

This review has some potential limitations. First, only peer-reviewed studies written in languages familiar to the research team were included, spanning from 2003 to 2023. As guidelines on gastroenteritis and home management have undergone changes in recent years, we believe that studies published more than 20 years ago are less applicable to the current context. Second, the search strategy employed in our systematic review did not include health care professionals in the search terms, potentially resulting in the omission of relevant articles. However, manual searches conducted in the literature did not yield additional studies beyond those already included in our systematic review. Lastly, the broad and subjective definition of enablers and barriers for home management has a degree of interpretive variability. To reduce this bias, data extraction and mapping them to the TDF was independently performed by 2 researchers and discussed within the research team in case of discrepancies.

### Quality Assessment

Most of the included studies (15/16) had a low risk of bias. Upon evaluating study design, most of the enablers and barriers as perceived by health care professionals were drawn from quantitative studies, whereas a predominant proportion of caregivers’ perspectives stemmed from qualitative studies. In terms of level of evidence, quantitative studies possess a greater rating if performed correctly.^50^ Noyes et al concluded that combining quantitative and qualitative evidence within reviews can offer enhanced insight into understanding complex interventions and underlying implementation systems.^51^ Nonetheless, as qualitative studies exploring health care professionals’ view are missing, further research is needed in this area.

## Conclusions

Optimizing home management for children with acute gastroenteritis requires the engagement of both health care professionals and caregivers. Various domains of the TDF and components of the COM-B explain the enablers and barriers that influence the home management. Among health care professionals, the greatest enabler lies within the “opportunity” component (ie, clinical decision tools, protocols, provision of free ORT), followed by their “capability” component (ie, knowledge about guidelines, ORT and ondansetron) to initiate home management. Conversely, caregivers’ factors rely more on internal factors within the “motivation” component (ie, emotions, insecurity, need for reassurance), where “opportunity” components (ie, information sheets, monitoring calls) could assist them in managing their child with gastroenteritis at home. By addressing these aspects, an effective strategy for optimizing home management for children with acute gastroenteritis could be established, potentially allowing more children to be treated at home.

## Data Statement

Data sharing statement available at www.jpeds.com.

## CRediT authorship contribution statement

**Anouk A.H. Weghorst:** Conceptualization, Data curation, Formal analysis, Funding acquisition, Methodology, Writing – original draft. **Joanna Lawrence:** Data curation, Formal analysis, Investigation, Writing – review & editing. **Danielle E.M.C. Jansen:** Conceptualization, Supervision, Writing – review & editing. **Gea A. Holtman:** Conceptualization, Writing – review & editing. **Lena A. Sanci:** Conceptualization, Supervision, Writing – review & editing. **Marjolein Y. Berger:** Conceptualization, Supervision, Writing – review & editing. **Harriet Hiscock:** Conceptualization, Supervision, Writing – review & editing.

## Declaration of Competing Interest

This research was supported by the 10.13039/501100001722KNAW Ter Meulen Grant/10.13039/501100001722KNAW Medical Sciences Fund, 10.13039/501100001722Royal Netherlands Academy of Arts & Sciences (KNAWWF/1085/TMB424). The funding source had no role in the design and conduct of the study; collection, management, analysis, and interpretation of the data; preparation, review or approval of the manuscript; and decision to submit the manuscript for publication. A.W. reports financial support was provided by Royal Netherlands Academy of Arts and Sciences. If there are other authors, they declare that they have no known competing financial interests or personal relationships that could have appeared to influence the work reported in this paper. There are no potential conflicts of interest to disclose.

## Supplementary Data

Appendix

Data Statement
