# Supplementary material for: Enablers and Barriers to Home Management for Children with Gastroenteritis: Systematic Review
Source: J Pediatr Clin Pract. 2024 May 15;14:200115. doi: 10.1016/j.jpedcp.2024.200115 (PMC11824653; doi:10.1016/j.jpedcp.2024.200115)
Supplement: Appendix [file mmc1.docx]

**Supplemental File 1. Search strategies by data source**

PubMed

1 "Gastroenteritis"[Mesh:NoExp] OR "Gastritis"[Mesh] OR Gastroenteritis[tiab] OR Gastroenteritides[tiab] OR Gastritis[tiab]

2 "Child"[Mesh] OR "Infant"[Mesh] OR child*[tiab] OR infan*[tiab] OR pediatr*[tiab] OR paediatr*[tiab] OR school*[tiab] OR preschool*[tiab] OR toddler*[tiab] OR kids[tiab] OR neonat*[tiab] OR young adult*[tiab] OR early life[tiab] OR early in life[tiab] OR early age[tiab] OR younger age[tiab] OR young age[tiab] OR "Family"[Mesh] OR parent*[tiab] OR mother*[tiab] OR father*[tiab] OR caregiver*[tiab] OR famil*[tiab] OR grandparent*[tiab]

3 "Home Care Services"[Mesh] OR home[tiab] OR homes[tiab] OR homecare*[tiab] OR "Self Care"[Mesh] OR “self care”[tiab] OR house*[tiab] OR "Patient Care Management"[Mesh] OR "preadmission management"[tiab] OR "nursing management"[tiab] OR "Fluid Therapy"[Mesh] OR fluid*[tiab] OR ors[tiab] OR oral rehydration*[tiab] OR advice*[tiab] OR educat*[tiab] OR inform*[tiab] OR "Diet"[Mesh] OR diet*[tiab] OR safety net*[tiab] OR antibiot*[tiab] OR "Antiemetics"[Mesh] OR antiemetic[tiab]

4 "Norway" OR "Switzerland" OR "Ireland" OR "Hong-Kong" OR "Iceland" OR "Germany" OR "Sweden" OR "Australia*" OR "Netherland*" OR "Dutch" OR "Denmark" OR "Singapore" OR "Finland" OR "United-Kingdom" OR "New-Zealand" OR "Belgium" OR "Canada" OR "United-States" OR "Austria" OR "Liechtenstein" OR "Japan" OR "Israel" OR "Slovenia" OR "Luxembourg" OR "South-Korea" OR "Andorra" OR "Latvia" OR "Portugal" OR "Slovakia" OR "Spain" OR "France" OR "Czech*" OR "Malta" OR "Italy" OR "Estonia" OR "United-Arab-Emirates" OR "Greece" OR "Cyprus" OR "Lithuania" OR "Poland" OR "UK" OR "US" OR "USA" OR "UAE" OR "NZ" OR "Greenland" OR "United-States" OR "Hong-Kong" OR "HK" OR "Croatia" OR "developed-countr*" OR "developed-nation*" OR "industrialized-countr*" OR "industrialized-nation*" OR "industrialised-countr*" OR "industrialised-nation*"

5 booksdocs[Filter] OR casereports[Filter] OR comment[Filter] OR editorial[Filter] OR guideline[Filter] OR letter[Filter] OR practiceguideline[Filter] OR preprint[Filter]

(1 AND 2 AND 3 AND 4) NOT 5

Filter: from 2003-3000/12/12

Embase (via Ovid)

1 gastritis/ or acute gastroenteritis/ or gastroenteritis/ or viral gastroenteritis/

2 (Gastroenteritis or gastroenteritides or gastritis).tw,kf,dq.

3 (Child* or infan* or pediatr* or paediatr* or school* or preschool* or toddler* or kids or neonat* or young-adult* or early-life or early-in-life or early-age or younger-age or young-age or Family or parent* or mother* or father* or caregiver* or famil* or grandparent*).tw,kf,dq,hw.

4 exp home care/

5 exp self care/

6 exp patient care/

7 exp fluid therapy/

8 exp diet/

9 exp antiemetic agent/

10 (home or homes or homecare* or self-care or house* or Patient-Care-Management or preadmission-management or nursing-management or fluid* or ors or oral-rehydration* or advice* or educat* or inform* or diet* or safety-net* or antibiot* or antiemetic).tw,kf,dq.

11 exp developed country/

12 (Norway or Switzerland or Ireland or Hong-Kong or Iceland or Germany or Sweden or Australia* or Netherland* or Dutch or Denmark or Singapore or Finland or United-Kingdom or New-Zealand or Belgium or Canada or United-States or Austria or Liechtenstein or Japan or Israel or Slovenia or Luxembourg or South-Korea or Andorra or Latvia or Portugal or Slovakia or Spain or France or Czech* or Malta or Italy or Estonia or United-Arab-Emirates or Greece or Cyprus or Lithuania or Poland or UK or US or USA or UAE or NZ or Greenland or United-States or Hong-Kong or HK or Croatia or developed-countr* or developed-nation* or industrialized-countr* or industrialized-nation* or industrialised-countr* or industrialised-nation*).tw,kf,dq,hw.

13 (1 or 2) and 3 and (4 or 5 or 6 or 7 or 8 or 9 or 10) and (11 or 12)

14 case report/

15 limit 14 to (conference abstract or conference paper or "conference review" or editorial or letter or "preprint (unpublished, non-peer reviewed)")

16 13 not (14 or 15)

17 limit 16 to yr="2003 -Current"

Web of Science

1 Gastroenteritis OR gastroenteritides OR gastritis

2 Child* OR infan* OR pediatr* OR paediatr* OR school* OR preschool* OR toddler* OR kids OR neonat* OR young-adult* OR early-life OR early-in-life OR early-age OR younger-age OR young-age OR Family OR parent* OR mother* OR father* OR caregiver* OR famil* OR grandparent*

3 home OR homes OR homecare* OR self-care OR house* OR Patient-Care-Management OR preadmission-management OR nursing-management OR fluid* OR ors OR oral-rehydration* OR advice* OR educat* OR inform* OR diet* OR safety-net* OR antibiot* OR antiemetic

4 Norway OR Switzerland OR Ireland OR Hong-Kong OR Iceland OR Germany OR Sweden OR Australia* OR Netherland* OR Dutch OR Denmark OR Singapore OR Finland OR United-Kingdom OR New-Zealand OR Belgium OR Canada OR United-States OR Austria OR Liechtenstein OR Japan OR Israel OR Slovenia OR Luxembourg OR South-Korea OR Andorra OR Latvia OR Portugal OR Slovakia OR Spain OR France OR Czech* OR Malta OR Italy OR Estonia OR United-Arab-Emirates OR Greece OR Cyprus OR Lithuania OR Poland OR UK OR US OR USA OR UAE OR NZ OR Greenland OR United-States OR Hong-Kong OR HK OR Croatia OR developed-countr* OR developed-nation* OR industrialized-countr* OR industrialized-nation* OR industrialised-countr* OR industrialised-nation*

#1 AND #2 AND #3 AND #4 and 2003-2023

CINAHL

1 (MH "Gastroenteritis") OR (MH "Gastritis")

2 Gastroenteritis or gastroenteritides or gastritis

3 Child* or infan* or pediatr* or paediatr* or school* or preschool* or toddler* or kids or neonat* or young-adult* or early-life or early-in-life or early-age or younger-age or young-age or Family or parent* or mother* or father* or caregiver* or famil* or grandparent*

4 (MH "Home Health Care+")

5 (MH "Self Care+")

6 (MH "Patient Care+")

7 (MH "Fluid Therapy+")

8 (MH "Diet+")

9 (MH "Antiemetics+")

10 home or homes or homecare* or self-care or house* or Patient-Care-Management or preadmission-management or nursing-management or fluid* or ors or oral-rehydration* or advice* or educat* or inform* or diet* or safety-net* or antibiot* or antiemetic

11 (MH "Developed Countries")

12 Norway or Switzerland or Ireland or Hong-Kong or Iceland or Germany or Sweden or Australia* or Netherland* or Dutch or Denmark or Singapore or Finland or United-Kingdom or New-Zealand or Belgium or Canada or United-States or Austria or Liechtenstein or Japan or Israel or Slovenia or Luxembourg or South-Korea or Andorra or Latvia or Portugal or Slovakia or Spain or France or Czech* or Malta or Italy or Estonia or United-Arab-Emirates or Greece or Cyprus or Lithuania or Poland or UK or US or USA or UAE or NZ or Greenland or United-States or Hong-Kong or HK or Croatia or developed-countr* or developed-nation* or industrialized-countr* or industrialized-nation* or industrialised-countr* or industrialised-nation*

13 (s1 or s2) and s3 and (s4 or s5 or s6 or s7 or s8 or s9 or s10) and (s11 or s12)

Limit 2003 – present; peer reviewed
