# Supplementary material for: Enablers and Barriers to Home Management for Children with Gastroenteritis: Systematic Review
Source: J Pediatr Clin Pract. 2024 May 15;14:200115. doi: 10.1016/j.jpedcp.2024.200115 (PMC11824653; doi:10.1016/j.jpedcp.2024.200115)
Supplement: Data Statement [file mmc2.docx]

Data Statement

All data will be available upon reasonable request.
